# Bufalin Inhibits HCT116 Colon Cancer Cells and Its Orthotopic Xenograft Tumor in Mice Model through Genes Related to Apoptotic and PTEN/AKT Pathways

**DOI:** 10.1155/2015/457193

**Published:** 2015-12-07

**Authors:** Jie Wang, Chao Chen, Shiying Wang, Yong Zhang, Peihao Yin, Zhongxiang Gao, Jie Xu, Dianxu Feng, Qinsong Zuo, Ronghua Zhao, Teng Chen

**Affiliations:** Department Surgery, Putuo Hospital, University of Traditional Chinese Medicine in Shanghai, Shanghai 200062, China

## Abstract

*Aims*. To investigate the anticolorectal cancer (CRC) effects of Bufalin, a bioactive polyhydroxysteroid from Venenum Bufonis, using HCT116 human CRC cell and an established orthotopic xenograft model in mice, and to explore the mechanisms of action. *Material and Methods*. Cultured HCT116 cells or BALB/c mice with orthotopic tumor were treated by Bufalin (positive control: 5-FU). Cell proliferation, apoptosis, and cycling were determined by MTT, Annexin V/PI staining, and flow cytometry, respectively. In mice, tumor inhibition rate and animal survival were calculated. The expressions of PTEN/phosphate-PTEN, AKT/phosphate-AKT, Bad, Bcl-xl, Bax, or Caspase-3 in cells and/or tumors were determined by Western blot or immunohistochemical staining. *Results*. Bufalin significantly inhibited cell proliferation and induced cell apoptosis and cycle arrest in a dose/time-dependent manner. In the animal model, Bufalin treatment resulted in significant inhibition of tumor growth and prolonged survival. In the Bufalin-treated cultured cells and/or xenograft tumors, the expressions of PTEN, Bad, Bax, and Caspase-3 were significantly increased, while p-AKT and Bcl-xL significantly decreased. *Conclusions*. Our results indicate that Bufalin inhibit cell proliferation and orthotopic tumor growth by inducing cell apoptosis through the intrinsic apoptotic pathway, which is of pivotal significance in the identification of an anticancer drug that may synergize with Bufalin.

## 1. Introduction

Colorectal cancer (CRC) is one the most common malignancies with an estimated over 1.2 million new diagnosed cases and 608,700 deaths worldwide each year [1]. Outcomes for patients with advanced CRC remain poor, with the median survival still less than 20 months [2]. Such a high incidence and the continued relatively poor prognosis of CRC underscore the need for a new therapy strategy.

Bufalin is a bioactive polyhydroxysteroid isolated from Venenum Bufonis, also called Chansu, a traditional Chinese medicine obtained from the skin and parotid venom glands of toads [3, 4]. Chansu, initially recorded more than 1000 years ago, is a well-known traditional Chinese medicine widely used in cancer treatment in China [5, 6]. Recent experimental studies have indicated that Chansu and its active compound Bufalin exhibited significant antitumor activity in various tumor models. The underlying mechanisms include the inhibition of cell proliferation, induction of cell differentiation and apoptosis, disruption of the cell cycle, inhibition of angiogenesis, reversal of multidrug resistance, and regulation of the immune response [7–11].

It was recently demonstrated that Bufalin causes apoptosis of CRC cells by inhibition of the Jak-STAT3 pathway [12]. However, there are few reports related to the anticancer effects of Bufalin in CRC animal models, and the exact mechanisms mediating such anticancer effects remain to be elucidated. This study aimed to investigate the* in vitro* and* in vivo* anticolon cancer effect of Bufalin using the HCT116 human colon carcinoma cell culture system and orthotopic xenograft CRC model. A secondary aim was to determine the possible mechanisms of action of Bufalin, focusing on apoptosis and the genes of apoptosis-related pathways.

## 2. Materials and Methods

### 2.1. Cell Culture and Animal Model

HCT116 colon cancer cells (Shanghai Institutes for Biological Sciences) were cultured in RPMI-1640 medium supplemented with 10% FBS (Life Technologies, Gibco USA), 100 units/mL penicillin, 100 *μ*g/mL streptomycin, and 2 m ML glutamine at 37°C with 5% CO_2_ and passaged using 0.25% trypsin/0.02% ethylenediamine tetraacetic acid (EDTA) upon 70% to 80% confluence (2-3 days). The maximal times of cell passage were less than 10 (<3 months) after the cells were recovered from frozen stocks. Cells in logarithmic growth phase were collected for the experiments.

For the* in vivo* studies, 5-6-week-old male BALB/c mice with a body weight of 18–20 grams were used (Shanghai Sippr BK Laboratory Animals Ltd., Shanghai, China). The mice were housed in a temperature and humidity controlled pathogen-free animal facility and fed according to the standard instruction provided by the animal provider. All animal studies were performed in accordance with the International Standards of Animal Welfare and were approved by the Institute of Animal Care and Use Committee of Shanghai University of Traditional Chinese Medicine (approval number: SYXK, Shanghai, 2013-0055).

The orthotopic HCT116 xenograft model was established as follows: HCT116 cells were harvested from the culture flask and suspended in culture medium to the concentration of 10 × 10^7^ mL^−1^. For each of the 5 mice, 200 *μ*L cell suspension was injected subcutaneously into the right axillary region. After two weeks, the subcutaneous xenograft tumors were harvested, cut into pieces (1.5 mm in diameter) after scraping off the surrounding fibrous capsule, and directly implanted into the axillary region subcutaneously for the next generation tumor mode. Tumors from the third subcutaneous generation were used to establish the orthotopic xenograft model, according to previous reports [13–16]. In brief, the third generation subcutaneous tumors were harvested and cut into pieces (1.5 mm in diameter), nude mice were anesthetized, and the abdomen was sterilized with iodine and alcohol swabs. A small midline incision was made and the caecum part of the intestine was exteriorized. Serosa of the site where tumor pieces were to be implanted was removed with an amyxis. Single pieces of the tumor were then implanted into the wall of caecum and fixed with medical glue. The intestine was returned to the abdominal cavity, and the abdominal wall was closed with surgical sutures. Then animals were kept in a sterile environment.

### 2.2. MTT (3-[4,5-Dimethylthiazol-2-yl]-2,5-diphenyl Tetrazolium Bromide) Assay

HCT116 cells were seeded at a concentration of 1 × 10^5^/well in 96-well plates and incubated for 24 h. Cells were then treated with Bufalin (Sigma-Aldrich, USA) at concentrations of 6, 3, 1, 0.6, 0.3, 0.1, 0.06, and 0.03 *μ*M for 24 h and 48 h. The negative control was treated with the culture median only. MTT assay was performed with a Cell Growth Determination Kit (Sigma-Aldrich, USA), according to the manufacturer's instructions. The cell growth inhibitory rate was calculated.

### 2.3. Flow Cytometric Analysis of Cell Cycle

HCT116 cells were seeded in the 6-well cell culture plates at a concentration of 2 × 10^5^ per well for 24 h and exposed to Bufalin at various concentrations of 0.03, 0.3, and 3 *μ*M for an additional 48 h. The negative control was treated with the culture median only. The cells were then collected, centrifuged, and fixed with 70% ethanol at 4°C, overnight. Finally, the fixed cells were stained by propidium iodide (PI) solution (1 mg/mL PI, 100 *μ*L per well) at room temperature for 30 minutes in a dark environment. Cell cycle was analyzed using flow cytometry.

### 2.4. Apoptosis Determination

Apoptosis for cells was determined by Annexin V and PI (V/PI) double staining. HCT116 cells were placed in the 6 well cell culture plates at a concentration of 2 × 10^5^ per well. After 24 h incubation, the cells were exposed to Bufalin at various concentrations of 0.03, 0.3, and 3 *μ*M and cultured for 48 h. Wells treated with culture median without Bufalin served as the negative control. Cells were then harvested and centrifuged, and the cell concentration was adjusted to 10^9^/L. The Annexin V-FITC/PI Apoptosis Detection Kit was used to stain the cells and cell apoptosis was determined by flow cytometry, according to manufacturer's instruction.

TUNEL assay (terminal deoxynucleotidyl transferase-mediated deoxy-UTP-fluorescein nick end labeling assay) was performed to determine apoptosis in the tissue samples. Four *μ*m of tissue sections was tested using the* In Situ* Cell Death Detection Kit (Roche, Mannheim, Germany) according to the procedures described by the manufacturer. Under high microscope, 5 randomly chosen fields (×400) without any necrotic areas were observed, and the average percentage of positive cells in the 5 fields for each section was calculated.

### 2.5. Morphologic Observation of Apoptotic Cells under Transmission Electron Microscope

HCT116 cells were seeded in the 6-well cell culture plates at a concentration of 2 × 10^5^ per well and incubated for 24 h. The cells were then treated with Bufalin at various concentrations of 0.03, 0.3, and 3 *μ*M (the negative wells were treated with culture medium without Bufalin) for another 24 h. Cells were collected and washed with Hanks solution followed by centrifuge. The samples were fixed in 2.5% glutaraldehyde (overnight), followed by 1% OsO4 for 1 h. Finally, cells were dehydrated in acetones of series concentrations and embedded in resin. Ultrathin sections were cut and observed under transmission electron microscope (Philips Tecnai-12).

#### 2.5.1. Western Blot

The expressions of Bad, PTEN/p-PTEN (phosphate PTEN), or AKT/p-AKT (phosphate AKT) in cells, and Bcl-xl, Bax, or Caspase-3 in xenograft tissue samples were determined by Western blot. Cultured HCT116 cells were placed in the 6 well cell culture plates at a concentration of 1 × 10^8^ cells per well and incubated for 24 h. The cells were then treated by Bufalin at various concentrations of 0.03, 0.3, and 3 *μ*M (negative control wells were treated by culture medium only) for 48 h. Total protein was extracted and purified by use of ProteoJET Mammalian Cell Lysis Reagent Kit (Thermo Scientific, USA). For xenograft tumor tissues, 50 mg of tissues was ground with liquid nitrogen and the protein was then extracted and purified using the ProteoJET Mammalian Cell Lysis Reagent Kit (Thermo Scientific, USA). Forty micrograms of protein extracted from each sample was mixed with a gel loading buffer, boiled for 5 min, separated on 8% SDS-polyacrylamide gel by electrophoresis, and then transferred to polyvinylidene difluoride membranes. To reduce the nonspecific background, the membranes were soaked in 5% skimmed milk powder solution at room temperature for 1 h. The blotted membranes were incubated with anti-Bad, anti-PTEN, anti-p-PTEN, anti-AKT, or anti-p-AKT monoclonal antibodies (Cell Signaling Technologies, USA) for the cultured cells and with anti-Bcl-xl, anti-Bax, or anti-Caspase-3 for the xenograft tissues, at 4°C overnight. Subsequently, the membranes were incubated with HRP-labeled anti-rabbit or anti-mouse antibodies at room temperature for 1 h. Bands were visualized by employing the ECL Plus Detection System (Millipore, Germany). Protein expression levels were quantified by Fluor Chem FC2 (Alpha Innotech, USA) and represented as the densitometric ratio of the targeted protein to the internal control, *β*-actin. All tests were performed in triplicate.

### 2.6. *In Vivo* Efficacy Studies

To validate the success of orthotopic xenograft, exploratory laparotomy was performed on 5 randomly chosen mice day 12 after tumor inoculation. Sixty mice with orthotopic xenograft tumor were randomly divided into five groups (12 mice in each group): NS group (treated with 0.2 mL normal saline), 5-Fu group (treated with 5-FU, 25 mg/kg), and three separate Bufalin groups treated with either low (0.5 mg/kg), medium (1.0 mg/kg), or high (1.5 mg/kg) doses of Bufalin. NS, 5-FU, and Bufalin were administrated by intraperitoneal injection, once per day from day 15 to day 21.

All mice were observed and weighed once per day during the study period. Three days after the last treatment, 6 mice from each group were euthanized. All the tumors were carefully resected and measured to obtain the maximum diameter (*a*) and minimum diameter (*b*). The tumor volume (*V*) and growth inhibition rate (IR) were calculated as follows: volume = *a* ^2^ *b*/2; IR = (*V*_control_ − *V*_treat_) × 100%/*V*_control_. All the tumors were bisected, one part was fixed in 10% formalin and paraffin embedded for routine HE and immunohistochemical staining, and the other was snap frozen and stored in liquid nitrogen for the TUNEL assay and Western blot. For the remaining 6 mice in each group, the time (survival) from tumor implantation to reaching moribund state was recorded.

### 2.7. Immunohistochemical (IHC) Staining

Serial sections of 4 *μ*m in thickness were cut from the formalin fixed and paraffin embedded orthotopic xenograft tissue samples for IHC staining. After retrieval of the antigen using citrate buffer (0.01 m ML, pH 6.0), the slides were washed three times with PBS and incubated in 10% normal goat serum to block nonspecific background staining. Sections were then incubated with rabbit anti-human Bcl-xl, Bax, Caspase-3, Bad, PTEN, or p-AKT antibodies (Cell signaling, USA) at 4°C overnight. After being washed three times with PBS, the sections were incubated with horseradish peroxidase- (HRP-) anti-rabbit IgG (Maixin Bio, Fuzhou, China) at room temperature for 30 min and finally washed with PBS and developed using diaminobenzidine (DAB). Five high power microscopic fields (×400) were randomly chosen from each slide to determine the positive staining intensity of Bcl-xl, Bax, Caspase-3, Bad, PTEN, or p-AKT protein by IPP software (Image-Pro Plus6.0, Media, Cybernetics). An unstained region was selected and set as the background. The expression level of the proteins was presented as the average staining intensity of the 5 fields from each slide.

### 2.8. Statistical Analysis

All data were expressed as percentage, mean with standard deviation (x ± sd), or median with 95% confidence interval (95% CI). Statistical analysis was done using Student's* t*-test, ANOVA, Chi square test, Mann-Whitney* U* test, Wilcoxon test, LSD with Games-Howell test, or Kaplan-Meier with Log-Rank test as appropriate, using the SPSS 17.0 for Windows. *P* < 0.05 was considered to be a statistically significant level.

## 3. Results

### 3.1. Effect of Bufalin on HCT116 Cell Proliferation and Cell Cycle

**Figure 1:**
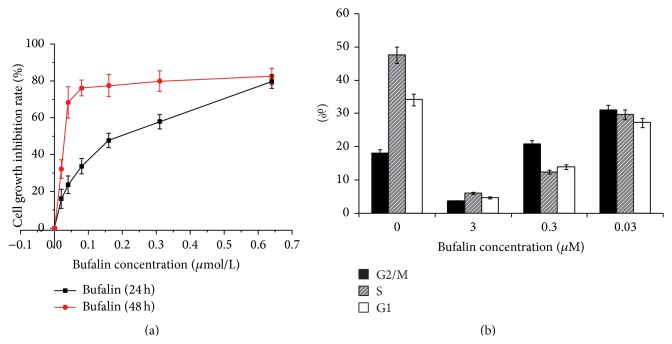
Effects of Bufalin on cell growth and cycle. (a) MTT (3-[4,5-dimethylthiazol-2-yl]-2,5-diphenyl tetrazolium bromide) assay showed Bufalin inhibited the cell growth with time- and dose-dependent manner. (b) Flow cytometric assay revealed that the number of G2/M cells was significantly increased in the Bufalin-treated cells in a dose-dependence manner compared to the controls (*P* < 0.05).

MTT tests showed that Bufalin inhibited cell proliferation. The IC_50_ at 24 and 48 h were 0.243 *μ*M and 0.024 *μ*M, respectively. The inhibitory effect was time- and dose-dependent (Figure 1). Flow cytometry revealed that the number of G_2_/M cells was significantly increased in those treated with 0.03, 0.3, and 3 *μ*M Bufalin in a dose-dependence manner, as compared to the controls (*P* < 0.05) (Figure 1).

### 3.2. Effect of Bufalin on HCT116 Cell Apoptosis and Morphology

**Figure 2:**
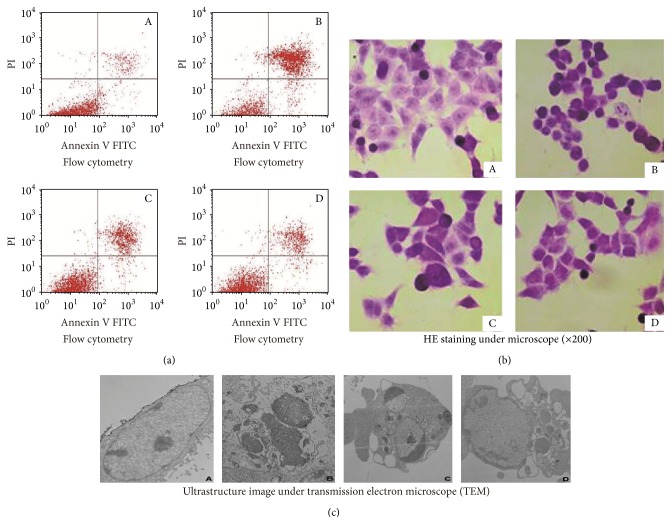
Effect of Bufalin on cell apoptosis. Flow cytometry on Annexin V/PI double stained cells showed that the apoptosis rates in the Bufalin-treated cells were significantly higher than that in the control group (*P* < 0.01) with dose-dependence. HE stained untreated cells appeared to be irregular spindle or polygon with cell mitosis (A), while the morphological changes of Bufalin-treated cells included cell dwindlement and shrinkage (C, D) or the spherical cell shape (B) and enlarged intercellular space (C, D) or disappeared connection (B) between the cells, chromatin condensation, and decreased mitosis. The ultrastructure changes included plasmatic rarefaction and chromatin margination (D), cytoplasmic scattering (C), chromatin condensation (C), and apoptotic body (B, C). Bufalin concentration: A: control, B: 3 *μ*M, C: 0.3 *μ*M, and D: 0.03 *μ*M.

V/PI double staining and flow cytometric analysis showed that the apoptosis rates in the cells treated with different doses of Bufalin were significantly higher than in the control group (*P* < 0.01) (Figure 2). The apoptosis rate was dependent on the doses of Bufalin (*P* < 0.01) (Figure 2). In addition, there was a significant difference noted among all groups treated with Bufalin (*P* < 0.05) (Figure 2).

Microscopically, the HE stained untreated cells appeared to be irregular spindle or polygon with cell mitosis. In the cells treated with 0.03 *μ*M Bufalin, the morphological changes included cell dwindlement and shrinkage, enlarged intercellular space, chromatin condensation, and decreased mitosis. The number of cells, however, was not significantly different compared to the controls. For cells treated with 0.3 and 3 *μ*M Bufalin, more condensed chromatin, disconnection between the cells, and loss of the spherical cell shape were seen (Figure 2); the number of cells was also significantly reduced compared to the controls (*P* < 0.05). Under transmission electron microscope, cells treated with 0.03 *μ*M Bufalin had typical ultramicrostructure changes of early stage apoptosis, including plasmatic rarefaction and chromatin margination. Cytoplasmic scattering, chromatin condensation, and an apoptotic body were seen in cells treated with 0.3 *μ*M Bufalin. An apoptotic body was also seen in cells treated with 3 *μ*M Bufalin (Figure 2).

### 3.3. Effect of Bufalin on the Expression of Bad, Caspase-3, PTEN/p-PTEN, or AKT/p-AKT in Cells

**Figure 3:**
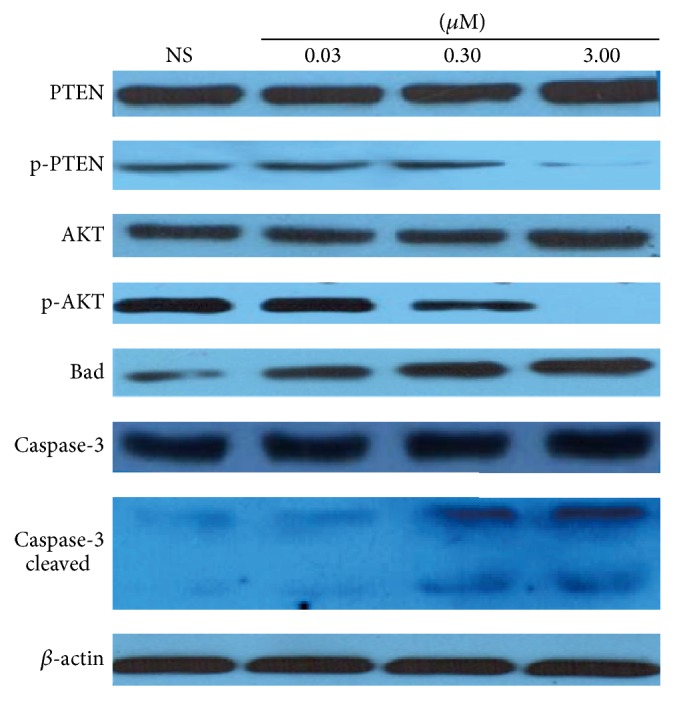
Expression of PTEN/p-PTEN, AKT/p-AKT, Bad, Caspase-3/Caspase-3 cleaved in control, and Bufalin-treated cells. The expression of PTEN was upregulated in cells treated with 3 *μ*M Bufalin, while the p-PTEN were significantly decreased in cells treated with 0.3 *μ*M and 3 *μ*M Bufalin compared to the control group (*P* < 0.05). There was significant downregulation of p-AKT in cells treated with 0.3 *μ*M and 3 *μ*M Bufalin (*P* < 0.05). The Bad expression was increased (*P* < 0.05) in Bufalin-treated cells. The treatment of 0.3 *μ*M and 3 *μ*M Bufalin caused cleaves of Caspase-3 (fragments with molecular weight of 17 or 19 kd).

The expression of PTEN was upregulated in cells treated with 3 *μ*M Bufalin, while the p-PTEN was significantly decreased in cells treated with 0.3 *μ*M and 3 *μ*M Bufalin compared to the control group (*P* < 0.05) (Figure 3). There was also an increase seen in Bad expression (*P* < 0.05) (Figure 3). There was no significant change of AKT expression in all groups treated with Bufalin, but a significant downregulation of p-AKT was seen in cells treated with 0.3 *μ*M and 3 *μ*M Bufalin (*P* < 0.05) (Figure 3). With an increased Bufalin dose, the phosphorylation of Caspase-3 was enhanced, and the treatment of 0.3 *μ*M and 3 *μ*M Bufalin caused cleaves of Caspase-3 (fragments with a molecular weight of 17 or 19 kd).

### 3.4. Efficacy of Bufalin on Orthotopic Xenograft Tumor in Mice

On day 12 after tumor inoculation, orthotopic xenograft tumor was seen in all 5 randomly chosen mice that underwent exploratory laparotomy and measured 0.8–1.0 cm in diameter. During the experiment, there were no significant clinical findings or body weight changes seen in all groups treated with Bufalin.

**Figure 4:**
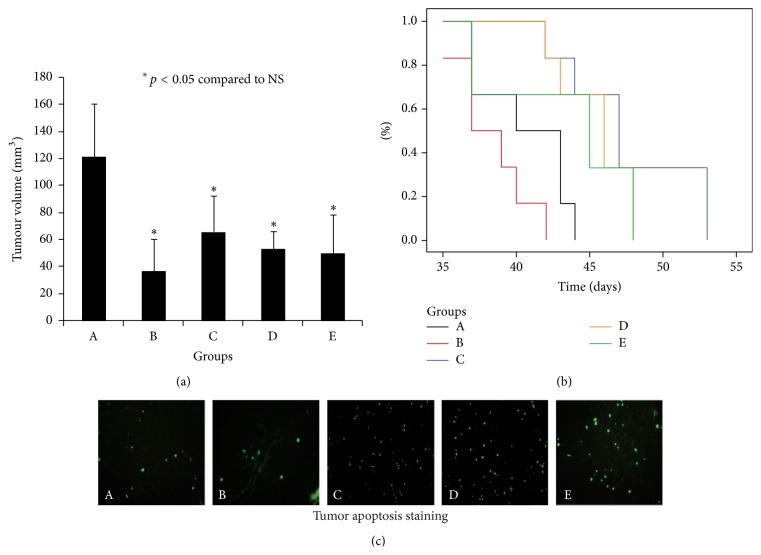
Effect of Bufalin on tumor volume, animal survival, and apoptosis. Bufalin treatment significantly inhibited tumor growth compared to the control group (*P* < 0.05) (a), prolonged survival time (*P* < 0.01) (b), and increased apoptosis in tumor (c). Groups: A: control, B: 5-FU, C: 0.5 mg/kg Bufalin, D: 1.0 mg/kg Bufalin, and E: 1.5 mg/kg Bufalin.

The inhibitory rates of 5-FU and Bufalin of low, medium, and high dosages were 69.6%, 45.6%, 56.2%, and 58.5%, respectively. The tumor volume was significantly lower in the treatment groups than in the control group (*P* < 0.05) (Table 1 and Figure 4). Mice treated with Bufalin showed a significantly prolonged survival time compared to the controls (*P* < 0.01) (Table 2 and Figure 4).

### 3.5. Effect of Bufalin on Tumor Apoptosis and Apoptosis Related Gene Expression

The apoptosis rate of xenograft tumor in each group is listed in Table 1. The tumor apoptotic rate in each Bufalin-treated group was significantly higher than in the control and 5-Fu groups (*P* < 0.05). There was no significant difference in the apoptotic rate between the 5-FU treated and the control groups. Figure 4 shows the apoptotic cells (cell nuclear stained by green fluorescence) under microscopy.

**Figure 5:**
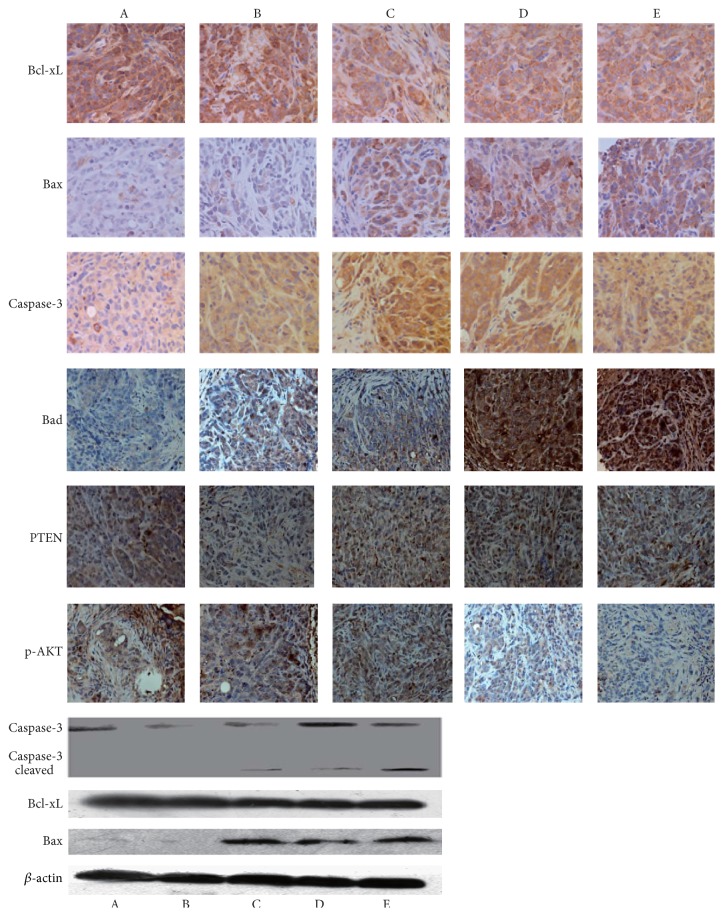
Immunohistochemical staining of Bcl-xL, Bax, Caspase-3, Bad, PTEN, pAKT, and Western blot test on selected proteins (Caspase-3/cleaved, Bcl-XL, and Bax). Upper: immunohistochemical staining (×400). Left: Western blot. Groups: A: NS, B: 5-FU, C: 0.5 mg/kg, D: 1.0 mg/kg, and E: 1.5 mg/kg.

Western blot showed that the expression of Bcl-xL in each Bufalin-treated group was significantly downregulated in a dose-dependent manner (*P* < 0.05) (Figure 5) compared to the control group. However, there was no difference in Bcl-xL expression among the three Bufalin-treated groups (*P* < 0.05) (Figure 5). The expression of Bax and Caspase-3 was increased in tumors treated with Bufalin, which was also dose-dependent (Figure 5). The expression of cleaved Caspase-3 was found in the three treated groups (Figure 5). IHC staining of the tumor section revealed similar results (Figure 5).

Moreover, the expression of Bad, PTEN, and p-AKT in tumor was detected by ICH (Figure 5). The results showed the Bad, PTEN, and p-AKT proteins were located in the cytoplasm. Bufalin induced the expression of Bad in a dose-dependent manner and was negative in the control group and weak, medium, and strong positive staining were seen in the groups treated with low, medium, and high dose Bufalin. The expression of PTEN was weak in the control group but was significantly increased when tumor was treated with Bufalin at doses of 1.0 and 1.5 mg/kg (*P* < 0.05). The protein of p-AKT was highly expressed in the control tumor and decreased after treatment with 0.5 mg/kg Bufalin, although this did not reach statistical significance. Tumors treated with 1.0 mg/kg and 1.5 mg/kg Bufalin had significantly reduced expression of p-AKT, compared to controls (*P* < 0.05).

## 4. Discussion

Bufalin is a major bioactive component of Venenum Bufonis and is extracted from Chansu the dried secretion from the skin of* Bufo bufo gargarizans* Cantor or* B. melanostictus* Schneider. It is the most active ingredient in Cinobufacini injection, which has been used in China for the treatment of liver, lung, and colorectal malignancies [5, 6]. Recently, results from several experimental studies have suggested that Bufalin exhibits significant antitumor activity in various cancer cells, such as human hepatocellular carcinoma [7], lung cancer [8], promyelocytic leukemia [17], and gastric cancer [10]. Consistent with the study by Qiu et al. [18], our study found that Bufalin reduces the viability of cultured HCT116 human CRC cells in a dose- and time-dependent manner. Similar results were reported in SW620 colon cancer cells [12].

Moreover, Bufalin was found to cause cell cycle arrest at M-phase (gastric cancer [10]), G1-phase (non-small-cell lung cancer A549 [8]), and G2/M-phase (leukemic cells [19] and osteosarcoma cells [20]). Bufalin decreases the proportion of ovarian cancer cells in the S-phase and increases the proportion in the G0/G1 phases of the cell cycle but has little effect on normal human endometrial epithelial cells [21]. In the present study, we found that Bufalin treatment caused cells to be accumulated at G2/M phase. Different changes in cell cycles for different cell lines suggest that the effects of Bufalin on cell cycles may be cell-type specific. It has also been found that Bufalin significantly induces HCT116 cell apoptosis, in keeping with previous reports on various cancers including liver [7], lung [10], prostate [11], and colorectum [12].

Encouraged by these findings, we aimed to assess the anticancer effects of Bufalin on a CRC animal model. For CRC, the subcutaneous xenograft model has been widely used to investigate the anticancer effects of the candidate drugs [22–25]. Such models, however, may cause changes in the biological characteristics of the xenograft tumor; for example, it may never lead to tumor metastasis, because of the inappropriate growth environment. Since Bufalin was been reported to inhibit cancer metastasis [18], studying the anticancer effect of Bufalin in an orthotopic animal model of CRC may be more appropriate compared to the traditional subcutaneous model. To our knowledge, this is the first study addressing the antitumor effect of Bufalin on a previously established orthotopic CRC xenograft model [26]. Bufalin has been found to significantly inhibit tumor growth and prolong survival in this animal model.

The underlying mechanisms mediating the anticancer effects of Bufalin are multifaceted and include autophagy, angiogenesis, and expression of genes related to the malignant phenotype in human cancer cells [7–11]. We have further explored the potential mechanisms by testing the expression of several critical genes of AKT-related apoptotic pathway including PTEN/p-PTEN, Bad, Bax, Bcl-XL, Caspase-3, and AKT/p-AKT on Bufalin-treated HCT116 cells and/or orthotopic xenograft tumors.

Human mammalian cells exhibit intrinsic and extrinsic pathways of apoptosis. In the intrinsic or mitochondrial pathway, the Bcl-2 family plays a crucial role in the control of apoptosis and can be classified into two functionally distinct groups: proapoptotic proteins such as Bax, Bad, and Bid and antiapoptotic proteins such as Bcl-2 and Bcl-xL, tightly regulating the mitochondrial apoptosis pathway. Bufalin can trigger mitochondria-mediated apoptosis in many cancer cells through downregulation of Bcl-2/Bcl-xL and/or upregulation of Bax/Bad/Bid [10, 21, 27, 28]. For example, Qi et al. [28] found that Bufalin can increase the expression of Fas, Bax, and Bid and reduce Bcl-2 expression, so as to disrupt the mitochondrial membrane potential indicating that Bufalin induces apoptosis through mitochondria-mediated pathways. Similarly, we found the increased expression of Bad in both Bufalin-treated cultured HCT116 cells and xenograft tumor tissues and increased expression of Bax and decreased expression of Bcl-xL in Bufalin-treated xenograft tumor tissues. Again this suggests that the mitochondria-mediated apoptosis plays a critical role.

We also studied the changes of upstream genes (PTEN and AKT) and downstream gene Caspase-3 in Bufalin-treated cultured cells and xenograft tumor tissue and found that PTEN expression increased while p-AKT decreased. PTEN is a tumor suppressing gene with many cancer-related biological functions such as inhibition of cell proliferation [29] and migration [30] and nuclear localization of Cyclin D1 [31]. With the deficiency and mutation of PTEN, multiple tumors may occur, including endometrial cancer [32], bladder cancer [33], and gastrointestinal cancer [34]. Nuclear PTEN expression gradually decreases after malignant transformation, and loss of PTEN expression in the nucleus is associated with tumor progression and poor clinical outcome in CRC [35]. Since PTEN has been reportedly implicated in the inactivation of PI3-K signaling [36], the Bufalin-related increase of PTEN may inactivate AKT by decreasing the p-AKT level through the PI-3K pathway. This is supported by the finding of decreased expression of p-AKT in both Bufalin-treated cells and xenograft tumors. Previous studies have shown that Akt inactivates Bad by phosphorylation and, consequently, acts upstream of mitochondria to prevent Cytochrome C release [37]. Akt also exerts its antiapoptotic effects in cells at a premitochondrial stage, at least in part, by inhibiting Bax conformational change and its redistribution to the mitochondrial membranes. In addition, Akt suppresses cell death by maintaining mitochondrial integrity and inhibiting Caspase activation [38]. Therefore, AKT inactivation may also play an important role on the proliferation and apoptosis of Bufalin-treated cells. Finally, as noted above PTEN-AKT-Bad/Bax signaling leads to the activation of the downstream protein, Caspase-3. We found that Caspase-3 was activated in Bufalin-treated cells and xenograft tumor. In this study, p-PTEN was surprisingly decreased in Bufalin-treated cells, which remains to be further elucidated.

Study limitations include the following: (1) only a single cell line was studied; (2) although we did find an association between Bufalin treatment and apoptosis-related gene expression, the detailed activation/inactivation process needs to be further elucidated by use of special knock-in or knock-down techniques, which is part of our ongoing research.

## 5. Conclusions

The results of this study indicate that Bufalin can significantly inhibit cell proliferation in cultured cells and tumor growth in the orthotopic xenograft animal model. Bufalin induced cell apoptosis through the activation of genes of intrinsic apoptotic pathways including PTEN, AKT, Bad/Bax, and Caspase-3, which is of pivotal significance in identifying anticancer drugs that may synergize with Bufalin treatment.

## Figures and Tables

**Table 1:** Tumor volumes, inhibitory rate, and apoptotic rate in xenograft tumor.

Group	*N*	Tumor volume (mm^3^)	Inhibitory rates (%)	Apoptosis rate (%)
NS	6	120.99 ± 39.69	—	3.46 ± 0.24
5-FU	6	36.81 ± 23.99^*∗*^	69.6	3.14 ± 0.56
BL	6	65.78 ± 26.14^*∗*^	45.6	11.93 ± 5.21^▲★^
BM	6	52.99 ± 13.45^*∗*^	56.2	13.07 ± 4.36^▲★^
BH	6	50.17 ± 28.12^*∗*^	58.5	9.61 ± 7.17^▲★^

*Note.* BL: Bufalin low dose group; BM: Bufalin median dose group; BH: Bufalin high dose group.

^*∗*^ *P* < 0.01 compared with NS group; ^★^ *P* < 0.01 and ^▲^ *P* < 0.05 compared with 5-FU group.

**Table 2:** Survival time of mice with xenograft tumor.

Group	*N*	Median survive (day)	95% CI^*∗*^	*P* (compare to control)
NS	6	40	35.2–44.8	—
5-FU	6	37	33.9–40.2	>0.05
BL	6	47	43.6–50.4	<0.05
BM	6	46	42.6–49.4	<0.05
BH	6	45	35.9–54.1	<0.05

*Note.* BL: Bufalin low dose group; BM: Bufalin median dose group; BH: Bufalin high dose group.

^*∗*^95% confidence interval.
